# Association between intrapartum fetal pulse oximetry and adverse perinatal and long-term outcomes: a systematic review and meta-analysis protocol

**DOI:** 10.12688/hrbopenres.13802.1

**Published:** 2023-10-20

**Authors:** Jill M. Mitchell, Siobhan Walsh, Laura J. O'Byrne, Virginia Conrick, Ray Burke, Ali S. Khashan, John Higgins, Richard Greene, Gillian M. Maher, Fergus P. McCarthy

**Affiliations:** 1 INFANT Research Centre, University College Cork, Cork, County Cork, Ireland; 2 Department of Obstetrics and Gynaecology, University College Cork, Cork, County Cork, Ireland; 3 UCC Library, University College Cork, Cork, County Cork, Ireland; 4 Tyndall National Institute, University College Cork, Cork, County Cork, Ireland; 5 School of Public Health, University College Cork, Cork, County Cork, Ireland; 6 National Perinatal Epidemiology Centre, University College Cork, Cork, County Cork, Ireland

**Keywords:** Labour, intrapartum, fetal monitoring, oximetry, oxygen saturation, blood gas monitoring, Sp02

## Abstract

**Background:** Current methods of intrapartum fetal monitoring based on heart rate, increase the rates of operative delivery but do not prevent or accurately detect fetal hypoxic brain injury. There is a need for more accurate methods of intrapartum fetal surveillance that will decrease the incidence of adverse perinatal and long-term neurodevelopmental outcomes while maintaining the lowest possible rate of obstetric intervention. Fetal pulse oximetry (FPO) is a technology that may contribute to improved intrapartum fetal wellbeing evaluation by providing a non-invasive measurement of fetal oxygenation status.

**Objective:** This systematic review and meta-analysis aims to synthesise the evidence examining the association between intrapartum fetal oxygen saturation levels and adverse perinatal and long-term outcomes in the offspring.

**Methods:** We will include randomised control trials (RCTs), cohort, cross-sectional and case-control studies which examine the use of FPO during labour as a means of measuring intrapartum fetal oxygen saturation and assess its effectiveness at detecting adverse perinatal and long-term outcomes compared to existing intrapartum surveillance methods. A detailed systematic search of PubMed, EMBASE, CINAHL, The Cochrane Library and Web of Science will be conducted following a detailed search strategy until August 2023. Three authors will independently review titles, abstracts and full text of articles. Two reviewers will independently extract data using pre-defined data extraction and assess the quality using the Risk of Bias tool for RCTs and Newcastle-Ottawa Scale for observational studies. We will use random-effects meta-analysis for each exposure-outcome association to calculate pooled estimates using the generic variance method. This systematic review will follow the Preferred Reporting Items for Systematic reviews and Meta-analyses guidelines.

**PROSPERO registration:** CRD42023457368 (04/09/2023)

## Introduction

Intrapartum fetal monitoring aims to improve perinatal outcome while avoiding unnecessary operative interventions ^1^. The gold standard for assessment of fetal well-being continues to be the auscultation of the fetal heart, along with the interpretation of alterations in the fetal heart rate pattern, as demonstrated through cardiotocography (CTG) to try to predict babies at risk of hypoxic brain injury and adverse perinatal outcomes. Despite its status as the established benchmark in care, employed in approximately 90% of births in the United States (US) and on a global scale, there is a pervasive consensus that current fetal monitoring devices based on heart rate, do not prevent or accurately detect fetal hypoxic brain injury ^2– 7^. The current use of cardiotocography (CTG) as a method of monitoring intrapartum fetal well‐being during labour is associated with an increased caesarean section rate, compared with intermittent auscultation of the fetal heart rate, resulting in a reduction in neonatal seizures, although no differences in other neonatal outcomes ^8^. CTG is complicated by significant inter- and intra-observer variation ^2, 9, 10^. Furthermore, CTG demonstrates a low positive predictive value for fetal hypoxia, meaning that among those fetuses that CTG indicates are at risk for hypoxia, a smaller proportion will truly be hypoxic ^2, 9, 10^. This can lead to an increased frequency of false-positive results, thus potentially prompting unnecessary interventions based on an overestimated risk of fetal hypoxia. Consequently, such misinterpretations could contribute to the escalating rates of caesarean deliveries observed worldwide. For instance, in the US, the caesarean delivery rate has surged, increasing from 20.7% in 1996 to 32.1% in 2021 ^11^. Similarly in the United Kingdom, the caesarean delivery rate has increased from 14.7% from 1990 to 1999 to 35.21% in 2021–2022 ^12, 13^.

Fetal blood sampling (FBS) is widely used as a complementary tool to improve the specificity and sensitivity of CTG ^14^. FBS has been shown to reduce operative vaginal delivery rates without affecting neonatal outcomes but it is a complex, invasive procedure ^2^. There is a need for more accurate methods of intrapartum fetal surveillance that will decrease the incidence of adverse neonatal and long-term neurodevelopmental outcomes while maintaining the lowest possible rate of obstetric intervention.

Fetal pulse oximetry (FPO) is a technology that may contribute to improved intrapartum fetal wellbeing evaluation by providing a non-invasive measurement of fetal oxygenation status ^15– 18^. FPO offers a potential dual advantage over traditional fetal heart rate monitoring. It quantifies the percentage of oxygenated haemoglobin directly, thereby assessing fetal oxygenation, which is pivotal in mediating the harmful consequences of hypoxia/ischaemia. Additionally, it utilises a well-established, non-invasive technology, regarded as safe and broadly implemented in all modern intensive care units and operating theatres ^8^. Based on data from both human and animal studies, average intrapartum fetal oxygen saturation (FSp02) range from 35% to 65% ^19– 21^. FSpO2 levels of 30% or higher are generally considered reassuring for the human fetus. However, if FSpO2 levels are less than 30% for a duration of 10 minutes or more, additional evaluation or intervention is warranted ^17, 19, 22– 24^ A study by McNamara *et al*. (n=100) demonstrated that babies born in poor condition had abnormal fetal oximetry values ^25^. FPO is advantageous over FBS in that it is a non-invasive and continuous monitoring technique.

A Cochrane Review published in 2014 compared fetal intrapartum pulse oximetry with other fetal surveillance techniques ^8^. This review included seven trials reporting on a total of 8013 pregnancies. The primary outcomes were caesarean section, hypoxic‐ischaemic encephalopathy, neonatal seizures and long‐term neurodevelopmental outcomes. The authors found no significant differences in the overall caesarean delivery rate between those monitored with FPO and those not monitored with FPO or for whom the FPO results were not displayed to the clinician or woman (four studies, n = 4008, risk ratio (RR) 0.99, 95% confidence intervals (CI) 0.86 to 1.13, I ^2^ = 45). The authors noted a reduction in the number of caesarean sections performed for cases of non-reassuring fetal status in the FPO plus CTG group compared to the CTG only group in two of the four analyses: firstly when considering pregnancies at or beyond 34 weeks where fetal FBS was not required prior to study entry (comprising four studies with 4008 participants, RR 0.65, 95% CI 0.46 to 0.90, I ^2^ = 63) Secondly, in situations where FBS was a prerequisite before study participation (one study involving 146 participants), the RR was notably low at 0.03, with a 95% confidence interval spanning from 0.00 to 0.44. Additionally, the review reported a reduction in operative births (comprising caesarean sections or operative vaginal births) for non-reassuring fetal status when FPO was combined with CTG monitoring, compared to CTG monitoring alone. This finding was consistent across two studies involving 1610 participants, with a RR of 0.74 (95% confidence interval: 0.62 to 0.89). However, no statistically significant differences were observed in several other outcome measures, including Apgar scores less than four at five minutes or less than seven at five minutes, umbilical arterial pH less than 7.10, neonatal intensive care unit (NICU) admissions, length of hospital stays, mortality, or fetal skin trauma, when comparing the use of FPO in conjunction with CTG to fetal electrocardiography combined with CTG. The results of this review may have been influenced by a large randomised control trial (RCT) by Bloom *et al*. ^26^ which had several limitations. Namely, that they did not describe the number of caesarean births indicated by the FPO results, nor did they describe counter-measures, including posture change according to the fetal oxygen saturation values, administration of tocolytic agents, and expedited delivery. Furthermore, observational studies were not included in the previous systematic review. In contrast, our planned review will take a more comprehensive approach, incorporating evidence not just from RCTs, but also from cohort studies, case-control studies, and observational studies.

Uchida *et al*. conducted a narrative review on FPO as a measure of intrapartum fetal condition. They concluded that FPO with fetal heart rate monitoring in selected cases of non-reassuring fetal status may reduce the caesarean section rate ^27^. While Uchida and colleagues discussed 31 studies and seven RCTs in their review of previous literature, they did not conduct a systematic review of previous literature. Therefore, we aimed to synthesise the previous literature examining the association between intrapartum fetal oxygen saturation and perinatal and long-term outcomes in the offspring in the form of a systematic review and meta-analysis.

This review has been registered with PROSPERO (CRD42023457368) on 4 ^th^ September 2023 and follows the PRISMA-P guidelines ^28^.

### Review question

Does low intrapartum fetal oxygen saturation as measured by fetal pulse oximetry increase the risk of adverse perinatal and long-term neurodevelopmental outcomes?

Does the addition of fetal pulse oximetry to established forms of intrapartum fetal monitoring such as cardiotocographs, improve perinatal and long-term neurodevelopmental outcomes?

## Methods

### Eligibility criteria

The following PICO criteria will guide this systematic review.

### Population

Women in labour with a cephalic, singleton live baby.

### Intervention

The use of fetal pulse oximetry during labour as a means of measuring intrapartum fetal oxygen saturation or low intrapartum fetal oxygen saturation in labour

### Comparison

We will compare the perinatal and long-term neurodevelopmental outcomes of offspring who had low oxygen saturations in labour versus those who had normal oxygen saturations in labour as measured by FPO or the use of FPO during labour as an adjunctive method of intrapartum surveillance compared to conventional fetal monitoring e.g. fetal heart rate monitoring by intermittent/continuous cardiotocography or the use of fetal scalp electrode, fetal blood sampling or electrocardiogram.

### Outcomes

Any measure of compromise of neonatal or childhood wellbeing - such as mortality; neonatal morbidity, including cord blood acidaemia, low APGAR scores, hypoxic ischaemic encephalopathy, intraventricular haemorrhage, seizures, periventricular leukomalacia, admission to the NICU and long term neurodevelopmental outcomes including cerebral palsy and developmental delay. See Table 1 for primary and secondary outcome measures.

**Table 1.** Review Outcome Measures.

Primary Outcomes	Perinatal Outcomes	Cord Blood acidaemia
		Abnormal Arterial or Venous Base Excess
		Abnormal Umbilical arterial or venous lactate levels
		Low 5 minute APGAR Score
		Admission to NICU
		Neonatal seizures
		Neonatal or intrapartum death
		Cardiopulmonary resuscitation or intubation required within 24 hours of life
		Hypoxic Ischaemic Encephalopathy
Secondary Outcomes		Low umbilical vein oxygen saturation
		Low umbilical artery oxygen saturation
		Operative Delivery for Non-Reassuring Fetal Status
		Operative Delivery for Dystocia
		Abnormal intrapartum fetal blood sample: scalp pH
		Abnormal intrapartum fetal blood sample: scalp lactate
	Long Term Outcomes	Cerebral Palsy
		Developmental Delay

### Studies

Randomised control trials and observational studies including cohort, case-control and cross-sectional studies

We will not exclude studies based on time frame or language. We will include published peer-reviewed studies only.

### Review exclusion criteria

1. Studies only available in abstract form.
2. Non-human studies.
3. Review articles, case reports, case series.
4. Conference proceedings, letters, commentaries, notes, editorials, dissertations.

### Literature search

We will use a two-part search strategy to identify studies meeting the inclusion criteria: (1) we will search electronic bibliographic databases for published work, using a comprehensive search strategy for fetal intrapartum pulse oximetry and perinatal and long-term outcomes; (2) we will hand-search the reference lists of studies included in the review and the reference lists of relevant, previously published reviews. The following electronic bibliographic databases will be searched: PubMed, EMBASE, CINAHL, The Cochrane Library and Web of Science. The search strategies for all databases can be found as *Extended data* ^29^.

### Study screening and selection

Titles and abstracts of the studies retrieved from each database search will be stored and managed in the Endnote reference manager and de-duplicated. Three independent reviewers (JM, SW, LOB) will screen all titles and abstracts. Full texts will be obtained where necessary to screen for eligibility. Where consensus on eligibility cannot be achieved, a fourth review author (FMcC) will be involved in the discussion.

### Data extraction and management

Two review authors (JM, SW) will independently extract data and discrepancies will be identified and resolved through discussion with a third author (FMcC), where necessary. A standardised, pre-piloted data extraction form will be used to extract data from the included studies. We will extract data including the author and year of publication, study design, country and setting of study, sample size, definition and or assessment of the exposures and outcome(s) of interest, comparison group, length of follow up, confounders adjusted for (if any), crude and adjusted estimates. If additional data is required from an eligible study, the corresponding author will be contacted via email. A reminder email will be sent two weeks later if the corresponding author does not reply.

### Quality appraisal of included studies

Articles which meet the selection criteria will be assessed for methodological quality independently by two reviewers using the Risk of Bias tool ^30^ for randomised controlled trials (RCT) and the Newcastle Ottawa Scale ^31^ for observational studies. Disagreements between the review authors over the quality assessment of each study will be resolved by discussion, with involvement of a third review author where necessary.

### Data synthesis, including assessment of heterogeneity

We will undertake separate meta-analyses for RCTs and observational studies using RevMan 5.4. We will also perform separate meta-analyses for each exposure-outcome associations. For example, low intrapartum fetal oxygen saturation and cord blood acidaemia, the addition of FPO as a monitoring method and cord blood acidaemia, low intrapartum fetal oxygen saturation and low 5 minute Apgar score and low intrapartum fetal oxygen saturation and admission to NICU. Heterogeneity will be assessed statistically using the I ^2^ statistic and also explored using subgroup analyses based on the different study designs included in this review. We will perform the subgroup/sensitivity analysis where the data allow, according to the study design (RCT, cohort, case-control and cross-sectional) and study quality/risk of bias (minimal/low versus moderate/high). We will perform a subgroup analysis using different cut-off values of cord blood acidaemia and FPO if different cut-offs are used in studies. Random effects meta-analyses will be performed to calculate overall pooled estimates where data allow. We will use the generic inverse variance method to display crude and adjusted results where possible. First, we will conduct a meta-analysis of all crude estimates for each exposure-outcome association. We will then conduct a meta-analysis of all adjusted estimates for each exposure-outcome association. We will consider any adjusted estimate as adjusted regardless of the variables adjusted for. When a meta-analysis cannot be conducted because of lack of data, a narrative synthesis of the results will be included.

The presence of publication bias will be evaluated using a funnel plot, provided a minimum inclusion of 10 studies or more in the meta-analysis. In instances where additional subgroup/sensitivity analyses are found within the meta-analysis, such as examinations to investigate potential high heterogeneity, these will be labelled as post-hoc analyses.

### Presenting and reporting the results

A PRISMA flow diagram will be incorporated to detail the sequential process of study selection, along with explanations for any studies excluded during the full-text review phase. Study characteristics and quality assessment of included studies will be displayed in tables, while pooled estimates will be presented using forest plots. Where data which is unsuitable for meta-analysis, results will be narratively synthesised.

## Conclusions

The systematic review and meta-analysis will summarise the existing literature investigating the association between intrapartum fetal oxygen saturation and adverse perinatal and long term outcomes in offspring. This review is of considerable importance as it explores the potential utility of fetal pulse oximetry as a method for intrapartum fetal monitoring. There is a pressing need for innovative and reliable approaches to monitor fetal well-being during labour, and this review could provide pivotal insights in this regard.

## Potential strengths and limitations of this study

The robustness of this review is bolstered by the implementation of a thorough search strategy, a prospectively registered protocol, and strict compliance with PRISMA guidelines. Additionally, the engagement of three reviewers in the process of eligibility screening and two reviewers in the process of data extraction, and quality assessment of the included studies serves to substantially mitigate the potential for reviewer-based bias in the systematic review. Furthermore, this review will not have language restrictions, reducing the risk that relevant indexed studies be overlooked. In the scope of this review, we will only incorporate studies that have been formally published. this may render our review susceptible to publication bias, as studies with significant or positive results are often likely to be published ^32^. If possible, a funnel plot will be used to assess the presence of publication bias. Moreover, the existence of confounding variables poses a significant challenge in observational research. Possible confounders might encompass the age of the mother, parity, maternal body mass index, heterogenous clinical approaches, different methods of monitoring FSp02 and pregnancy complications such as intrauterine growth restriction, pre-eclampsia and gestational diabetes mellitus. As noted previously, our meta-analyses will present both unadjusted and adjusted outcomes, whenever feasible, using the generic inverse variance approach. This adjustment will be based on the definitions provided in each of the studies we've reviewed.

## Dissemination

It is anticipated that findings of this review will be disseminated through publication in a peer-reviewed journal and presented at scientific conferences.

## Study status

Not commenced yet.

## Data Availability

No data are associated with this article. Fighsare: Search Strategy - Association between Intrapartum Fetal Pulse Oximetry and Adverse Perinatal and Long-term Outcomes- a Systematic Review and Meta-analysis Protocol.docx. *https://doi.org/10.6084/m9.figshare.24049890.v3* ^29^. Fighsare: PRISMA-P checklist for ‘Association between intrapartum fetal pulse oximetry and adverse perinatal and long-term outcomes: a systematic review and meta-analysis protocol. *https://doi.org/10.6084/m9.figshare.24049899* ^28^. Data are available under the terms of the Creative Commons Attribution 4.0 International license (CC-BY 4.0).
