# Association between intrapartum fetal pulse oximetry and adverse perinatal and long-term outcomes: a systematic review and meta-analysis protocol

**DOI:** 10.12688/hrbopenres.13802.2

**Published:** 2024-03-28

**Authors:** Jill M. Mitchell, Siobhan Walsh, Laura J. O'Byrne, Virginia Conrick, Ray Burke, Ali S. Khashan, John Higgins, Richard Greene, Gillian M. Maher, Fergus P. McCarthy

**Affiliations:** 1 INFANT Research Centre, University College Cork, Cork, County Cork, Ireland; 2 Department of Obstetrics and Gynaecology, University College Cork, Cork, County Cork, Ireland; 3 UCC Library, University College Cork, Cork, County Cork, Ireland; 4 Tyndall National Institute, University College Cork, Cork, County Cork, Ireland; 5 School of Public Health, University College Cork, Cork, County Cork, Ireland; 6 National Perinatal Epidemiology Centre, University College Cork, Cork, County Cork, Ireland

**Keywords:** Labour, intrapartum, fetal monitoring, oximetry, oxygen saturation, blood gas monitoring, Sp02

## Abstract

**Background:**

Current methods of intrapartum fetal monitoring based on heart rate, increase the rates of operative delivery but do not prevent or accurately detect fetal hypoxic brain injury. There is a need for more accurate methods of intrapartum fetal surveillance that will decrease the incidence of adverse perinatal and long-term neurodevelopmental outcomes while maintaining the lowest possible rate of obstetric intervention. Fetal pulse oximetry (FPO) is a technology that may contribute to improved intrapartum fetal wellbeing evaluation by providing a non-invasive measurement of fetal oxygenation status.

**Objective:**

This systematic review and meta-analysis aims to synthesise the evidence examining the association between intrapartum fetal oxygen saturation levels and adverse perinatal and long-term outcomes in the offspring.

**Methods:**

We will include randomised control trials (RCTs), cohort, cross-sectional and case-control studies which examine the use of FPO during labour as a means of measuring intrapartum fetal oxygen saturation and assess its effectiveness at detecting adverse perinatal and long-term outcomes compared to existing intrapartum surveillance methods. A detailed systematic search of PubMed, EMBASE, CINAHL, The Cochrane Library, Web of Science, ClinicalTrials.Gov and WHO ICTRP will be conducted following a detailed search strategy until February 2024. Three authors will independently review titles, abstracts and full text of articles. Two reviewers will independently extract data using a pre-defined data extraction form and assess the quality of included studies using the Risk of Bias tool for RCTs and Newcastle-Ottawa Scale for observational studies. The grading of recommendations, assessment, development, and evaluation (GRADE) approach will be used to evaluate the certainty of the evidence. We will use random-effects meta-analysis for each exposure-outcome association to calculate pooled estimates using the generic variance method. This systematic review will follow the Preferred Reporting Items for Systematic reviews and Meta-analyses and MOOSE guidelines.

**PROSPERO registration:**

CRD42023457368 (04/09/2023)

## Introduction

Intrapartum fetal monitoring aims to improve perinatal outcome while avoiding unnecessary operative interventions ^1^. The gold standard for assessment of fetal well-being continues to be the auscultation of the fetal heart, along with the interpretation of alterations in the fetal heart rate pattern, as demonstrated through cardiotocography (CTG) to try to predict babies at risk of hypoxic brain injury and adverse perinatal outcomes. Despite its status as the established benchmark in care, employed in approximately 90% of births in the United States (US) and on a global scale, there is a pervasive consensus that current fetal monitoring devices based on heart rate, do not prevent or accurately detect fetal hypoxic brain injury ^2– 7^. The current use of cardiotocography (CTG) as a method of monitoring intrapartum fetal well‐being during labour is associated with an increased caesarean section rate, compared with intermittent auscultation of the fetal heart rate, resulting in a reduction in neonatal seizures, although no differences in other neonatal outcomes ^8^. CTG is complicated by significant inter- and intra-observer variation ^2, 9, 10^. Furthermore, CTG demonstrates a low positive predictive value for fetal hypoxia, meaning that among those fetuses that CTG indicates are at risk for hypoxia, a smaller proportion will truly be hypoxic ^2, 9, 10^. This can lead to an increased frequency of false-positive results, thus potentially prompting unnecessary interventions based on an overestimated risk of fetal hypoxia. Consequently, such misinterpretations could contribute to the escalating rates of caesarean deliveries observed worldwide. For instance, in the US, the caesarean delivery rate has surged, increasing from 20.7% in 1996 to 32.1% in 2021 ^11^. Similarly in the United Kingdom, the caesarean delivery rate has increased from 14.7% from 1990 to 1999 to 35.21% in 2021–2022 ^12, 13^.

Fetal blood sampling (FBS) is widely used as a complementary tool to improve the specificity and sensitivity of CTG ^14^. FBS has been shown to reduce operative vaginal delivery rates without affecting neonatal outcomes but it is a complex, invasive procedure ^2^. There is a need for more accurate methods of intrapartum fetal surveillance that will decrease the incidence of adverse neonatal and long-term neurodevelopmental outcomes while maintaining the lowest possible rate of obstetric intervention.

Fetal pulse oximetry (FPO) is a technology that may contribute to improved intrapartum fetal wellbeing evaluation by providing a non-invasive measurement of fetal oxygenation status ^15– 18^. FPO offers a potential dual advantage over traditional fetal heart rate monitoring. It quantifies the percentage of oxygenated haemoglobin directly, thereby assessing fetal oxygenation, which is pivotal in mediating the harmful consequences of hypoxia/ischaemia. Additionally, it utilises a well-established, non-invasive technology, regarded as safe and broadly implemented in all modern intensive care units and operating theatres ^8^. Based on data from both human and animal studies, average intrapartum fetal oxygen saturation (FSp02) range from 35% to 65% ^19– 21^. FSpO2 levels of 30% or higher are generally considered reassuring for the human fetus. However, if FSpO2 levels are less than 30% for a duration of 10 minutes or more, additional evaluation or intervention is warranted ^17, 19, 22– 24^ A study by McNamara *et al*. (n=100) demonstrated that babies born in poor condition had abnormal fetal oximetry values ^25^. FPO is advantageous over FBS in that it is a non-invasive and continuous monitoring technique.

A Cochrane Review published in 2014 compared fetal intrapartum pulse oximetry with other fetal surveillance techniques ^8^. This review included seven trials reporting on a total of 8013 pregnancies. The primary outcomes were caesarean section, hypoxic‐ischaemic encephalopathy, neonatal seizures and long‐term neurodevelopmental outcomes. The authors found no significant differences in the overall caesarean delivery rate between those monitored with FPO and those not monitored with FPO or for whom the FPO results were not displayed to the clinician or woman (four studies, n = 4008, risk ratio (RR) 0.99, 95% confidence intervals (CI) 0.86 to 1.13, I ^2^ = 45). The authors noted a reduction in the number of caesarean sections performed for cases of non-reassuring fetal status in the FPO plus CTG group compared to the CTG only group in two of the four analyses: firstly when considering pregnancies at or beyond 34 weeks where fetal FBS was not required prior to study entry (comprising four studies with 4008 participants, RR 0.65, 95% CI 0.46 to 0.90, I ^2^ = 63) Secondly, in situations where FBS was a prerequisite before study participation (one study involving 146 participants), the RR was notably low at 0.03, with a 95% confidence interval spanning from 0.00 to 0.44. Additionally, the review reported a reduction in operative births (comprising caesarean sections or operative vaginal births) for non-reassuring fetal status when FPO was combined with CTG monitoring, compared to CTG monitoring alone. This finding was consistent across two studies involving 1610 participants, with a RR of 0.74 (95% confidence interval: 0.62 to 0.89). However, no statistically significant differences were observed in several other outcome measures, including Apgar scores less than four at five minutes or less than seven at five minutes, umbilical arterial pH less than 7.10, neonatal intensive care unit (NICU) admissions, length of hospital stays, mortality, or fetal skin trauma, when comparing the use of FPO in conjunction with CTG to fetal electrocardiography combined with CTG. The results of this review may have been influenced by a large randomised control trial (RCT) by Bloom *et al*. ^26^ which had several limitations. Namely, that they did not describe the number of caesarean births indicated by the FPO results, nor did they describe counter-measures, including posture change according to the fetal oxygen saturation values, administration of tocolytic agents, and expedited delivery. Furthermore, observational studies were not included in the previous systematic review. In contrast, our planned review will take a more comprehensive approach, incorporating evidence not just from RCTs, but also from cohort studies, case-control studies, and observational studies.

Uchida *et al*. conducted a narrative review on FPO as a measure of intrapartum fetal condition. They concluded that FPO with fetal heart rate monitoring in selected cases of non-reassuring fetal status may reduce the caesarean section rate ^27^. While Uchida and colleagues discussed 31 studies and seven RCTs in their review of previous literature, they did not conduct a systematic review of previous literature. Therefore, we aimed to synthesise the previous literature examining the association between intrapartum fetal oxygen saturation and perinatal and long-term outcomes in the offspring in the form of a systematic review and meta-analysis.

This review has been registered with PROSPERO (CRD42023457368) on 4 ^th^ September 2023 and follows the PRISMA-P and MOOSE guidelines ^28^.

### Review question

This review will aim to assess the association between intrapartum fetal oxygen saturation less than 30% and the risk of adverse perinatal and long-term neurodevelopmental outcomes. It also aims to investigate whether the addition of the measurement of fetal oxygen saturation by fetal pulse oximetry to established forms of fetal monitoring such as cardiotocographs can reduce the rate of operative delivery without affecting perinatal and long term behavioural outcomes.

## Methods

### Eligibility criteria

The following PICO criteria will guide this systematic review.

### Population

Women in labour with a cephalic baby.

Our search will not exclude multiple pregnancies although we don’t expect to find studies where FPO is measured in the non-presenting fetus as it is only technically possible to monitor fetal oxygen saturation using fetal pulse oximetry for the presenting twin using the available technology as the devices are positioned between the fetal cheek or scapula and uterine wall or placed on the fetal scalp ^29– 34^. This being said, a study is currently ongoing investigating the concurrence of internal and external fetal oxygen sensors using a novel transabdominal fetal pulse oximetry device ^35^. However, an inclusion criterion in this study is singleton pregnancy.

### Intervention/Exposure

1. Low fetal oxygen saturation defined as less than 30% (exposure).
2. The use of fetal pulse oximetry during labour as a means of measuring intrapartum fetal oxygen saturation (intervention).

### Comparison

1. Normal fetal oxygen saturation defined as greater than or equal to 30% (unexposed group).
2. Conventional fetal monitoring e.g. fetal heart rate monitoring by CTG or the use of fetal scalp electrode, fetal blood sampling or fetal electrocardiogram without the measurement of fetal oxygen saturation (control).

### Outcomes

The primary outcomes of this review are; umbilical artery pH less than 7.20, 7.15 and 7.0 and 5 minute APGAR Score less than 7. Secondary outcomes include; (i) umbilical artery base excess less then -10mmol/L, (ii) umbilical artery lactate greater than 4.8 mmol/L, correlation between FSp02 and umbilical artery and vein pH, (iii) admission to the neonatal intensive care unit (NICU), (iv) neonatal or intrapartum death, (v) cardiopulmonary resuscitation or intubation required within 24 hours of life, (vi) hypoxic ischaemic encephalopathy, (vii) umbilical vein oxygen saturation less than 55%, (viii) low umbilical artery oxygen saturation less than 30%, (ix) operative delivery for non-reassuring fetal status (as defined by local protocols in each study), (x) operative delivery for dystocia, (xi) fetal scalp pH <7.20, (xii) fetal scalp lactate >4.8mmol/L, (xiii) cerebral palsy and (xiv) severe neurodevelopmental disability. There is no consensus on the specific definition of developmental delay ^36^. Multiple different assessment tools exist and the recommended tools have changed over the past 30 years ^37, 38^. Severe neurodevelopmental disability was defined by a previous Cochrane review ^39^; “any one or a combination of the following: non‐ambulant cerebral palsy, developmental delay (developmental quotient less than 70), auditory and visual impairment assessed at 12 months of age or more. Development should have been assessed by means of a previously validated tool, such as Bayley Scales of Infant Development (Psychomotor Developmental Index and Mental Developmental Index ^40^. See Table 1 for primary and secondary outcome measures.

**Table 1.** Review Outcomes.

Primary Outcomes	Perinatal Outcomes	Umbilical artery pH less than 7.20
		Umbilical artery pH less than 7.15
		Umbilical artery pH less than 7.0
		5 minute APGAR Score less than 7
Secondary Outcomes		Umbilical artery base excess less then -10mmol/L
		Umbilical artery lactate greater than 4.8 mmol/L
		Correlation between FSp02 and umbilical artery and vein pH
		Admission to NICU
		Neonatal or intrapartum death
		Cardiopulmonary resuscitation or intubation required within 24 hours of life
		Hypoxic Ischaemic Encephalopathy
		Umbilical vein oxygen saturation less than 55%
		Low umbilical artery oxygen saturation less than 30%
		Operative Delivery for Non-Reassuring Fetal Status (as defined by local protocols in each study)
		Operative Delivery for Dystocia
		Fetal scalp pH <7.20
		Fetal scalp lactate >4.8mmol/L
	Long Term Outcomes	Cerebral Palsy
		Severe Neurodevelopmental Disability *

* *Perinatal outcomes are defined as outcomes occurring within the first 24 hours of life*. ** *Severe neurodevelopmental disability was defined by a previous Cochrane review ^39^; “any one or a combination of the following: non‐ambulant cerebral palsy, developmental delay (developmental quotient less than 70), auditory and visual impairment assessed at 12 months of age or more. Development should have been assessed by means of a previously validated tool, such as Bayley Scales of Infant Development (Psychomotor Developmental Index and Mental Developmental Index” ^40^*.

### Studies

Randomised control trials and observational studies including cohort, case-control and cross-sectional studies

We will not exclude studies based on time frame or language.

### Review exclusion criteria

- Studies only available in abstract form.
- Non-human studies.
- Review articles, case reports, case series.
- Conference proceedings, letters, commentaries, notes, editorials, dissertations.

### Literature search

We will use a two-part search strategy to identify studies meeting the inclusion criteria: (1) we will search electronic bibliographic databases and clinical trial registers for relevant work, using a comprehensive search strategy for fetal intrapartum pulse oximetry and perinatal and long-term outcomes; (2) we will hand-search the reference lists of studies included in the review and the reference lists of relevant, previously published reviews. The following electronic bibliographic databases will be searched: PubMed, EMBASE, CINAHL, The Cochrane Library and Web of Science ClinicalTrials.Gov and WHO ICTRP until February 2024.

The search strategies for all databases can be found as *Extended data* ^41^.

### Study screening and selection

Titles and abstracts of the studies retrieved from each database search will be stored and managed in the Endnote reference manager and de-duplicated. Three independent reviewers (JM, SW, LOB) will screen all titles and abstracts. Full texts will be obtained where necessary to screen for eligibility. Where consensus on eligibility cannot be achieved, a fourth review author (FMcC) will be involved in the discussion.

### Data extraction and management

Two review authors (JM, SW) will independently extract data and discrepancies will be identified and resolved through discussion with a third author (FMcC), where necessary. A standardised, pre-piloted data extraction form will be used to extract data from the included studies. We will extract data including the author and year of publication, study design, country and setting of study, sample size, definition and or assessment of the exposures and outcome(s) of interest, comparison group, length of follow up, confounders adjusted for (if any), crude and adjusted estimates. If additional data is required from an eligible study, the corresponding author will be contacted via email. A reminder email will be sent two weeks later if the corresponding author does not reply.

### Quality appraisal of included studies

Articles which meet the selection criteria will be assessed for methodological quality independently by two reviewers using the Risk of Bias tool ^42^ for randomised controlled trials (RCT) and the Newcastle Ottawa Scale ^43^ for observational studies. Disagreements between the review authors over the quality assessment of each study will be resolved by discussion, with involvement of a third review author where necessary. The grading of recommendations, assessment, development, and evaluation (GRADE) approach will be used to evaluate the certainty of the evidence by two reviewers independently (J.M. and S.W). The certainty of the evidence was assessed using the GRADEpro software on the basis of the following domains: the study design, risk of bias, imprecision, inconsistency, indirectness, and publication bias ^44^.

### Data synthesis, including assessment of heterogeneity

We will undertake separate meta-analyses for RCTs and observational studies using RevMan Web. We will also perform separate meta-analyses for each exposure-outcome associations. For example, intrapartum fetal oxygen saturation less than 30% and umbilical artery pH less than 7.20, the addition of FPO as a monitoring method and umbilical artery pH less than 7.0, intrapartum fetal oxygen saturation less than 30% and 5 minute Apgar score less than 7 and intrapartum fetal oxygen saturation less than 30% and admission to NICU. Random effects meta-analyses will be performed to calculate overall pooled estimates where data allow. We will use the generic inverse variance method to display crude and adjusted results where possible. First, we will conduct a meta-analysis of all crude estimates for each exposure-outcome association. We will then conduct a meta-analysis of all adjusted estimates for each exposure-outcome association. We will consider any adjusted estimate as adjusted regardless of the variables adjusted for. When a meta-analysis cannot be conducted because of lack of data, a narrative synthesis of the results will be included. The summary measures will be reported as odds ratio (OR) with 95% confidence interval (CI). We will present the correlation between FSp02 and umbilical artery and vein pH in narrative form.

Heterogeneity will be assessed statistically using the I ^2^ statistic and explored using subgroup analyses based on the different study designs included in this review. We will perform the subgroup/sensitivity analysis where the data allow, according to the study design (RCT, cohort, case-control and cross-sectional) and study quality/risk of bias (minimal/low versus moderate/high). Where the data allow, we will perform subgroup analyses based on gestational age. Furthermore, for meta-analyses which included at least three studies, 95% prediction intervals were calculated in Microsoft Excel using formulae previously outlined by Borenstein *et al.* ^45, 46^.

The presence of publication bias will be evaluated using a funnel plot, and by conducting an Egger’s test for asymmetry of the funnel plot, provided a minimum inclusion of 10 studies or more in the meta-analysis. In instances where additional subgroup/sensitivity analyses are found within the meta-analysis, such as examinations to investigate potential high heterogeneity, these will be labelled as post-hoc analyses.

### Presenting and reporting the results

A PRISMA flow diagram will be incorporated to detail the sequential process of study selection, along with explanations for any studies excluded during the full-text review phase. Study characteristics and quality assessment of included studies will be displayed in tables, while pooled estimates will be presented using forest plots. Where data which is unsuitable for meta-analysis, results will be narratively synthesised.

## Conclusions

The systematic review and meta-analysis will summarise the existing literature investigating the association between intrapartum fetal oxygen saturation and adverse perinatal and long term outcomes in offspring. This review is of considerable importance as it explores the potential utility of fetal pulse oximetry as a method for intrapartum fetal monitoring. There is a pressing need for innovative and reliable approaches to monitor fetal well-being during labour, and this review could provide pivotal insights in this regard.

## Potential strengths and limitations of this study

The robustness of this review is bolstered by the implementation of a thorough search strategy, a prospectively registered protocol, and strict compliance with PRISMA and MOOSE guidelines. Additionally, the engagement of three reviewers in the process of eligibility screening and two reviewers in the process of data extraction, and quality assessment of the included studies serves to substantially mitigate the potential for reviewer-based bias in the systematic review. Furthermore, this review will not have language restrictions, reducing the risk that relevant studies be overlooked. The existence of confounding variables poses a significant challenge in observational research. Possible confounders might encompass the age of the mother, parity, maternal body mass index, heterogenous clinical approaches, different methods of monitoring FSp02 and pregnancy complications such as intrauterine growth restriction, pre-eclampsia and gestational diabetes mellitus. As noted previously, our meta-analyses will present both unadjusted and adjusted outcomes, whenever feasible, using the generic inverse variance approach. This adjustment will be based on the definitions provided in each of the studies we've reviewed. Family wise error rate increases when comparing many secondary outcomes, therefore significant results will be interpreted with caution based on plausibility, theory and uncertainty.

## Dissemination

It is anticipated that findings of this review will be disseminated through publication in a peer-reviewed journal and presented at scientific conferences.

## Data Availability

No data are associated with this article. Fighsare: Search Strategy - Association between Intrapartum Fetal Pulse Oximetry and Adverse Perinatal and Long-term Outcomes- a Systematic Review and Meta-analysis Protocol.docx. *https://doi.org/10.6084/m9.figshare.24049890.v3* ^41^. Fighsare: PRISMA-P checklist for ‘Association between intrapartum fetal pulse oximetry and adverse perinatal and long-term outcomes: a systematic review and meta-analysis protocol. *https://doi.org/10.6084/m9.figshare.24049899* ^28^. Data are available under the terms of the Creative Commons Attribution 4.0 International license (CC-BY 4.0).
