# Supplementary material for: Association between intrapartum fetal pulse oximetry and adverse perinatal and long-term outcomes: a systematic review and meta-analysis protocol
Source: HRB Open Res. 2024 Mar 28;6:63. Originally published 2023 Oct 20. [Version 2] doi: 10.12688/hrbopenres.13802.2 (PMC11019289; doi:10.12688/hrbopenres.13802.2)
Supplement: Peer review history

# Reviewer report: Reviewer response for version 2

**DOI:** 10.21956/hrbopenres.15221.r38895

**Published:** 2024-04-15

**Authors:** James X Sotiropoulos, Sol Libesman

**Affiliations:** 1 Evidence Integration, The University of Sydney, Sydney, New South Wales, Australia

Thank you for the opportunity to comment on the revised manuscript.

 The authors have addressed all concerns in detail. I have no further comments.

 Best wishes with your study.

Is the study design appropriate for the research question?

Yes

Is the rationale for, and objectives of, the study clearly described?

Yes

Are sufficient details of the methods provided to allow replication by others?

Partly

Are the datasets clearly presented in a useable and accessible format?

Yes

Reviewer Expertise:

Systematic Reviews, Meta-analysis, Research Integrity, Neonatal and Perinatal Research

We confirm that we have read this submission and believe that we have an appropriate level of expertise to confirm that it is of an acceptable scientific standard.

# Reviewer report: Reviewer response for version 1

**DOI:** 10.21956/hrbopenres.15101.r37131

**Published:** 2023-11-27

**Authors:** James X Sotiropoulos, Sol Libesman

**Affiliations:** 1 Evidence Integration, The University of Sydney, Sydney, New South Wales, Australia

We thank you for the opportunity to review this study protocol. 

 We commend the authors for their practice of open science and publishing the protocol at this stage with pre-registration following PRISMA-P guidelines. We will focus mainly on the methods in this review, as it is most aligned with my experience, and provide some (hopefully) constructive feedback. 

**Introduction**

 The introduction is clear and describes the need for this study – with a recent narrative review without systematic review and meta-analysis indicating potential findings.  

**Methods**

 Note Table 1 formatting appears odd on the online system – please check 

 Are there any inclusion/exclusion criteria beyond cephalic, singleton live baby? 

 The authors' inclusion/exclusion criteria is singleton pregnancies only. Is there a good justification for this? If possible, it may provide future utility in collecting data for multiples, even if only reporting it in stratified analysis separately. If FPO is not feasible/reliable in multiple pregnancies this should be clearly stated and cited. 

 The authors could consider further specifying the outcomes. For example, the cut-off for “low”, and “abnormal” are not provided. Could you specify how developmental delay will be measured including a cut-off (if this has been established)? Could you also specify the time in which these outcomes are measured (e.g., the primary endpoints), the measurement units (if relevant), and the relative effects that will be used to compare the groups? 

 In the section “comparator” you mention “We will compare …offspring who had low oxygen saturations in labour versus those who had normal oxygen saturations in labour as measured by…”. Could you specify the cut-off you plan to use for low?  For further guidance on the description of treatments and outcomes, the ‘estimand’ framework may help you clarify elements of treatment, population, outcome, population level summaries, and more. 

 There are two review questions nominated, but the comparator section only appears to address the first review question.

 The authors plan to use I ^2^ as a measure of heterogeneity. I ^2^ is a flawed measure of heterogeneity, although it permeates systematic review and meta-analysis across all disciplines. Please see this article (https://doi.org/10.1002/jrsm.1230, ^1^). Will the authors consider, instead or in addition to I ^2^, graphically representing heterogeneity with prediction intervals or another measure of between-study variance? The Cochrane Handbook provides some useful guidance. 

 The authors have not included an assessment of the overall certainty of evidence (e.g., GRADE). This has also been marked as “NA” in the PRISMA-P document. I strongly recommend the use of GRADE to assess the overall certainty of evidence, or if it is to be omitted for a valid reason that this be explained in the methods and/or discussion. Alternatively, if it is omitted a clear plan as to how the evidence will be evaluated should be provided. 

 The authors have noted the potential impact of publication bias and plan to use a funnel plot. They may consider a contour-enhanced funnel plot, PET-PEESE, or p-curve analysis to examine publication bias. They might also consider a search of clinical trial registers to look for other registered studies that may be unpublished but have useable data for inclusion in meta-analysis. Guidance for searching clinical trial registers is here https://doi.org/10.1136/bmj-2021-068791 ^2^.

 The authors intend to search only until August 2023. By the time the results of this study are ready for publication the search may be outdated. I note the PROSPERO registration says the search will begin in 2024. This may be a typo. Otherwise, I suggest the search is updated beyond August 2023 prior to the results publication. 

 The authors have prespecified their outcomes, however, have listed many primary outcomes and stated “Any measure of compromise of neonatal or childhood wellbeing…” will be used. I suggest pre-specifying a single (or at most two) key primary outcomes, and other outcomes be listed as secondary. Mortality and neurodevelopment may be appropriate given the introduction; however, this is beyond my expertise and knowledge of the literature. I also suggest revising the sentence so that the measures of compromised are more specific, and not “any” measure is included. 

 There are many secondary outcomes. I would recommend a sentence acknowledging the increased family wise error rate when conducting multiple comparisons. E.g., “because family wise error rate increases when comparing many secondary outcomes, significant results will be interpreted with caution based on plausibility, theory and uncertainty.” 

**Discussion**

 Strengths and limitations are appropriately noted.

Is the study design appropriate for the research question?

Yes

Is the rationale for, and objectives of, the study clearly described?

Yes

Are sufficient details of the methods provided to allow replication by others?

Partly

Are the datasets clearly presented in a useable and accessible format?

Yes

Reviewer Expertise:

Systematic Reviews, Meta-analysis, Research Integrity, Neonatal and Perinatal Research

We confirm that we have read this submission and believe that we have an appropriate level of expertise to confirm that it is of an acceptable scientific standard, however we have significant reservations, as outlined above.

## References

[1] : Basics of meta-analysis: I2 is not an absolute measure of heterogeneity. *Res Synth Methods* .2017;8(1) : 10.1002/jrsm.1230 5-18 10.1002/jrsm.1230 28058794

[2] : Searching clinical trials registers: guide for systematic reviewers. *BMJ* .2022;377: 10.1136/bmj-2021-068791 e068791 10.1136/bmj-2021-068791 35473822

# Response

**Published:** 2024-03-13

**Authors:** Jill Mitchell

**Affiliations:** Obstetrics and Gynaecology, University College Cork, Cork, County Cork, Ireland

Dear Dr Sotiropoulos and Dr Libesman,

 We thank you both for your helpful review of our manuscript entitled “Association between Intrapartum Fetal Pulse Oximetry and Adverse Perinatal and Long-term Outcomes: a Systematic Review and Meta-analysis Protocol”.

 Please find below an itemized reply addressing each comment in your Reviewer’s Report.

**1.Introduction**

**The introduction is clear and describes the need for this study – with a recent narrative review without systematic review and meta-analysis indicating potential findings.** 

 We thank you for your positive comment regarding our Introduction. 

**2.Methods**

**Table 1**

 Table 1. Review Outcomes

*https://hrbopenresearch.s3.eu-west-1.amazonaws.com/linked/197134.13802-Author_response_to_Referee_James_X_Sotiropoulos_v1__2.pdf*

**3. Are there any inclusion/exclusion criteria beyond cephalic, singleton live baby? The authors' inclusion/exclusion criteria is singleton pregnancies only. Is there a good justification for this? If possible, it may provide future utility in collecting data for multiples, even if only reporting it in stratified analysis separately. If FPO is not feasible/reliable in multiple pregnancies this should be clearly stated and cited.**

 We have removed the word “singleton” from the inclusion criteria so that our search will include multiple pregnancies although we don’t expect to find studies where fetal oxygen saturation is measured in both twins as it is therefore only technically possible to monitor fetal oxygen saturation using fetal pulse oximetry for the presenting twin using the available technology. This being said, a study is currently ongoing investigating the concurrence of internal and external fetal oxygen sensors using a novel transabdominal fetal pulse oximetry device (3). However, an inclusion criteria in this study is singleton pregnancy.

 We have updated the manuscript to reflect this, see p9, lines 95 to 111.

 “ ***Population***

*Women in labour with a cephalic baby.*

*Our search will not exclude multiple pregnancies although we don’t expect to find studies where FPO is measured in the non-presenting fetus as it is only technically possible to monitor fetal oxygen saturation using fetal pulse oximetry for the presenting twin using the available technology as the devices are* *positioned between the fetal cheek or scapula and uterine wall or placed on the fetal scalp* *(4-9)* *. This being said, a study is currently ongoing investigating the concurrence of internal and external fetal oxygen sensors using a novel transabdominal fetal pulse oximetry device* *(3)* *. However, an inclusion criteria in this study is singleton pregnancy.”*

 We decided not to exclude based on gestational age as we want to include as many relevant studies as possible. Where the data allow, we will perform subgroup analyses based on gestational age. See p14, lines 257-260.

*“Where the data allow, we will perform subgroup analyses based on gestational age.”*

**4. The authors could consider further specifying the outcomes. For example, the cut-off for “low”, and “abnormal” are not provided.**

 Thank you for this suggestion. We have updated our protocol and Table 1 to further define the outcomes. See p10-11, lines 124-153;

*“The primary outcomes of this review are; (i) umbilical artery pH less than 7.20, (ii) umbilical artery pH less than 7.15 and (iii) umbilical artery pH less than 7.0, (iv) 5 minute APGAR Score less than 7. Secondary outcomes include; (i) umbilical artery base excess less then -10mmol/L, (ii) umbilical artery lactate greater than 4.8 mmol/L, (iii) admission to the neonatal intensive care unit (NICU), (iv) neonatal or intrapartum death, (v) cardiopulmonary resuscitation or intubation required within 24 hours of life, (vi) hypoxic ischaemic encephalopathy, (vii) umbilical vein oxygen saturation less than 55%, (viii) low umbilical artery oxygen saturation less than 30%, (ix) operative delivery for non-reassuring fetal status (as defined by local protocols in each study) (x) operative delivery for dystocia, (xi) fetal scalp pH <7.20, (xii) fetal scalp lactate >4.8mmol/L, (xiii) cerebral palsy and (xiv) severe neurodevelopmental disability. There is no consensus on the specific definition of developmental delay* *(10)* *. Multiple different assessment tools exist and the recommended tools have changed over the past 30 years* *(11, 12)* *. Severe neurodevelopmental disability was defined by a previous Cochrane review* *(1)* *; “any one or a combination of the following: non‐ambulant cerebral palsy, developmental delay (developmental quotient less than 70), auditory and visual impairment assessed at 12 months of age or more. Development should have been assessed by means of a previously validated tool, such as Bayley Scales of Infant Development (Psychomotor Developmental Index and Mental Developmental Index* *(2)* *.”*

 5. **Could you specify how developmental delay will be measured including a cut-off (if this has been established)?**

 We have considered your suggestion and with this in mind have decided to update our objective from “developmental delay” to “severe neurodevelopmental disability” as defined by a previous Cochrane review(1). This is outlined in our on p11, lines 146 to 153, as follows:

*“There is no consensus on the specific definition of developmental delay* *(10)* *. Multiple different assessment tools exist and the recommended tools have changed over the past 30 years* *(11, 12)* *. Severe neurodevelopmental disability was defined by a previous Cochrane review* *(1)* *; “any one or a combination of the following: non‐ambulant cerebral palsy, developmental delay (developmental quotient less than 70), auditory and visual impairment assessed at 12 months of age or more. Development should have been assessed by means of a previously validated tool, such as Bayley Scales of Infant Development (Psychomotor Developmental Index and Mental Developmental Index* *(2)* *.”*

**6. Could you also specify the time in which these outcomes are measured (e.g., the primary endpoints),the measurement units (if relevant), and the relative effects that will be used to compare the groups?**

 We have defined the term “perinatal outcomes” as outcomes occurring within the first 24 hours of life (see p19, Table 1, footnote 1) and severe neurodevelopmental disability was defined as any one or a combination of the following: non‐ambulant cerebral palsy, developmental delay (developmental quotient less than 70), auditory and visual impairment assessed at 12 months of age (see p19, Table 1, footnote 2).

 “* *Perinatal outcomes are defined as outcomes occurring within the first 24 hours of life.*

 ** *Severe neurodevelopmental disability was defined by a previous Cochrane review* *(1)* *; “any one or a combination of the following: non‐ambulant cerebral palsy, developmental delay (developmental quotient less than 70), auditory and visual impairment assessed at 12 months of age or more. Development should have been assessed by means of a previously validated tool, such as Bayley Scales of Infant Development (Psychomotor Developmental Index and Mental Developmental Index”* *(2)* *.”*

 The summary measures will be reported as odds ratio with 95% confidence interval. We will present the correlation between FSp02 and umbilical artery and vein pH in narrative form.

 This has been clarified on p14, lines 242 to 244.

*“The summary measures will be reported as odds ratio (OR) with 95% confidence interval (CI).* *We will present the correlation between FSp02 and umbilical artery and vein pH in narrative form.”*

**7. In the section “comparator” you mention “We will compare …offspring who had low oxygen saturations in labour versus those who had normal oxygen saturations in labour as measured by…”. Could you specify the cut-off you plan to use for low?  For further guidance on the description of treatments and outcomes, the ‘estimand’ framework may help you clarify elements of treatment, population, outcome, population level summaries, and more. There are two review questions nominated, but the comparator section only appears to address the first review question.**

 We have now edited our “intervention/exposure” and “comparison” to incorporate your suggestion. . This is outlined in our manuscript as follows (p10, lines 113 to 122):

***“Intervention/Exposure***

1. *Low fetal oxygen saturation defined as less than 30% (exposure).*
2. *The use of fetal pulse oximetry during labour as a means of measuring intrapartum fetal oxygen saturation (intervention). *

***Comparison***

1. *Normal fetal oxygen saturation defined as greater than or equal to 30% (unexposed group).”*
2. *Conventional fetal monitoring e.g. fetal heart rate monitoring by CTG or the use of fetal scalp electrode, fetal blood sampling or fetal electrocardiogram without the measurement of fetal oxygen saturation (control).*

**8. The authors plan to use I ^2^ as a measure of heterogeneity. I ^2^ is a flawed measure of heterogeneity, although it permeates systematic review and meta-analysis across all disciplines. Please see this article (https://doi.org/10.1002/jrsm.1230,** **^1^** **). Will the authors consider, instead or in addition to I ^2^, graphically representing heterogeneity with prediction intervals or another measure of between-study variance? The Cochrane Handbook provides some useful guidance.**

 Thank you for this comment. We have amended methods section to include prediction intervals where meta-analysis include three or more studies, see p14, lines 251 to 253.

*“Furthermore, for meta-analyses which include at least three studies, 95% prediction intervals will be calculated in Microsoft Excel using formulae previously outlined by Borenstein et al* *(13, 14)* *’*

**9. The authors have not included an assessment of the overall certainty of evidence (e.g., GRADE). This has also been marked as “NA” in the PRISMA-P document. I strongly recommend the use of GRADE to assess the overall certainty of evidence, or if it is to be omitted for a valid reason that this be explained in the methods and/or discussion. Alternatively, if it is omitted a clear plan as to how the evidence will be evaluated should be provided.**

 Thank you for this recommendation. We have decided to use an assessment of the overall certainty of evidence using the GRADE approach. This has been added to the manuscript. See p13, lines 216 to 220.

*“The grading of recommendations, assessment, development, and evaluation (GRADE) approach will be used to evaluate the certainty of the evidence for primary outcomes by two reviewers independently (J.M. and S.W). The certainty of the evidence was assessed using the GRADEpro software on the basis of the following domains: the study design, risk of bias, imprecision, inconsistency, indirectness, and publication bias* *(15)* *.”*

**10. The authors have noted the potential impact of publication bias and plan to use a funnel plot. They may consider a contour-enhanced funnel plot, PET-PEESE, or p-curve analysis to examine publication bias.**

 Thank you for this suggestion. We have decided to assess publication bias using Egger’s test in addition to funnel plots. See p14, lines 254 to 256.

*“The presence of publication bias will be evaluated using a funnel plot,* *and by conducting an Egger’s test for asymmetry of the funnel plot* *, provided a minimum inclusion of 10 studies or more in the meta-analysis.”*

**11. They might also consider a search of clinical trial registers to look for other registered studies that may be unpublished but have useable data for inclusion in meta-analysis. Guidance for searching clinical trial registers is here** **https://doi.org/10.1136/bmj-2021-068791****** **^2^** .

 We have decided to include clinical trial registers, namely ClinicalTrials.Gov and WHO ICTRP in our search. We have amended our manuscript to read (p12, lines 185 to 187);

*“The following electronic bibliographic databases and clinical trial registers will be searched: PubMed, EMBASE, CINAHL, The Cochrane Library, Web of Science, ClinicalTrials.Gov and WHO ICTRP until February 2024.”*

**12. The authors intend to search only until August 2023. By the time the results of this study are ready for publication the search may be outdated. I note the PROSPERO registration says the search will begin in 2024. This may be a typo. Otherwise, I suggest the search is updated beyond August 2023 prior to the results publication.** 

 Thank you for this suggestion. We have extended our search timeline to February 2024, see p12, lines 185 to 187.

*“The following electronic bibliographic databases and clinical trial registers will be searched: PubMed, EMBASE, CINAHL, The Cochrane Library, Web of Science, ClinicalTrials.Gov and WHO ICTRP until February 2024.”*

**13. The authors have prespecified their outcomes, however, have listed many primary outcomes and stated “Any measure of compromise of neonatal or childhood wellbeing…” will be used. I suggest pre-specifying a single (or at most two) key primary outcomes, and other outcomes be listed as secondary. Mortality and neurodevelopment may be appropriate given the introduction; however, this is beyond my expertise and knowledge of the literature. I also suggest revising the sentence so that the measures of compromised are more specific, and not “any” measure is included.** 

 Thank you for this comment. We have taken this suggestion on board and have updated our outcomes accordingly. See p10, lines 124 to 146.

*“The primary outcomes of this review are; (i) umbilical artery pH less than 7.20, (ii) umbilical artery pH less than 7.15 and (iii) umbilical artery pH less than 7.0, (iv) 5 minute APGAR Score less than 7. Secondary outcomes include; (i) umbilical artery base excess less then -10mmol/L, (ii) umbilical artery lactate greater than 4.8 mmol/L, (iii) admission to the neonatal intensive care unit (NICU), (iv) neonatal or intrapartum death, (v) cardiopulmonary resuscitation or intubation required within 24 hours of life, (vi) hypoxic ischaemic encephalopathy, (vii) umbilical vein oxygen saturation less than 55%, (viii) low umbilical artery oxygen saturation less than 30%, (ix) operative delivery for non-reassuring fetal status (as defined by local protocols in each study) (x) operative delivery for dystocia, (xi) fetal scalp pH <7.20, (xii) fetal scalp lactate >4.8mmol/L, (xiii) cerebral palsy and (xiv) severe neurodevelopmental disability.*

**14. There are many secondary outcomes. I would recommend a sentence acknowledging the increased family wise error rate when conducting multiple comparisons. E.g., “because family wise error rate increases when comparing many secondary outcomes, significant results will be interpreted with caution based on plausibility, theory and uncertainty.”**

 We have included this sentence and will also include it in the limitations section of our review. See p16, page 309 to 311.

*“Family wise error rate increases when comparing many secondary outcomes, therefore significant results will be interpreted with caution based on plausibility, theory and uncertainty.”*

**15.Discussion**

**Strengths and limitations are appropriately noted.**

 We thank you for your positive comment regarding our Discussion. 

 We hope these amendments address the suggestions raised. Should any further amendments be necessary, we would be happy to address them accordingly. 

 Sincerely,

 Dr Jill Mitchell

 Corresponding author.  

 Research Fellow

 Department of Obstetrics and Gynaecology, University College Cork, Cork, Ireland.

**References**

 1.         Devane D, Lalor JG, Daly S, McGuire W, Cuthbert A, Smith V. Cardiotocography versus intermittent auscultation of fetal heart on admission to labour ward for assessment of fetal wellbeing. Cochrane Database of Systematic Reviews. 2017(1).

 2.         Bayley N. Manual for the Bayley Scales of Infant Development 2nd Edition: San Antonio: The Psychological Corporation; 1993.

 3.         Rosen M. Trans-abdominal Fetal Pulse Oximetry. 2023.

 4.         Siristatidis C, Salamalekis E, Kassanos D, Loghis C, Creatsas G. Evaluation of fetal intrapartum hypoxia by middle cerebral and umbilical artery Doppler velocimetry with simultaneous cardiotocography and pulse oximetry. Archives of gynecology and obstetrics. 2004;270(4):265-70.

 5.         Siristatidis C, Salamalekis E, Vitoratos N, Loghis C, Salloum J, Kassanos D, et al. Intrapartum surveillance of IUGR fetuses with cardiotocography and fetal pulse oximetry. Biol Neonate. 2003;83(3):162-5.

 6.         Vitoratos N, Salamalekis E, Saloum J, Makrakis E, Creatsas G. Abnormal fetal heart rate patterns during the active phase of labor: The value of fetal oxygen saturation. Journal of Maternal-Fetal Medicine. 2002;11(1):46-9.

 7.         Carbonne B, Langer B, Goffinet F, Audibert F, Tardif D, Le Goueff F, et al. Multicenter study on the clinical value of fetal pulse oximetry: II. Compared predictive values of pulse oximetry and fetal blood analysis. American Journal of Obstetrics and Gynecology. 1997;177(3):593-8.

 8.         Luttkus AK, Callsen TA, Stupin JH, Dudenhausen JW. Pulse oximetry during labour - Does it give rise to hope? Value of saturation monitoring in comparison to fetal blood gas status. European Journal of Obstetrics and Gynecology and Reproductive Biology. 2003;110(SUPPL.):S132-S8.

 9.         Uchida T, Kanayama N, Mukai M, Furuta N, Itoh H, Suzuki H, et al. Examiner's finger-mounted fetal tissue oximetry: a preliminary report on 30 cases. Journal of Perinatal Medicine. 2016;44(7):745-9.

 10.       Petersen MC, Kube DA, Palmer FB. Classification of developmental delays. Seminars in Pediatric Neurology. 1998;5(1):2-14.

 11.       Rosenbaum P. Screening tests and standardized assessments used to identify and characterize developmental delays. Seminars in Pediatric Neurology. 1998;5(1):27-32.

 12.       Çelik P, Ayranci Sucakli I, Yakut HI. Which Bayley-III cut-off values should be used in different developmental levels? Turk J Med Sci. 2020;50(4):764-70.

 13.       Borenstein M, Higgins JP, Hedges LV, Rothstein HR. Basics of meta-analysis: I(2) is not an absolute measure of heterogeneity. Res Synth Methods. 2017;8(1):5-18.

 14.       Borenstein M, Hedges LV, Higgins JPT, Rothstein HR. Prediction Intervals.  Introduction to Meta‐Analysis2009. p. 127-33.

 15.       Balshem H, Helfand M, Schünemann HJ, Oxman AD, Kunz R, Brozek J, et al. GRADE guidelines: 3. Rating the quality of evidence. Journal of Clinical Epidemiology. 2011;64(4):401-6.

# Reviewer report: Reviewer response for version 1

**DOI:** 10.21956/hrbopenres.15101.r36909

**Published:** 2023-11-10

**Authors:** Maeve Eogan

**Affiliations:** 1 Rotunda Hospital, Dublin, Ireland; 2 Obstetrics and Gynecology, Royal College of Surgeons in Ireland Faculty of Sports and Exercise Medicine, Dublin, Leinster, Ireland

This protocol for a systematic review & metanalysis synthesising evidence on this relevant topic for parents and practitioners is already registered with Prospero, and is well summarised and detailed in the attached documents. The background to the topic is eloquently described, the review questions are distinct and the methods are described in sufficient detail. Inclusion and exclusion criteria for identified studies are prescribed, and the methodology and proposed reporting of findings appear appropriate.

Is the study design appropriate for the research question?

Yes

Is the rationale for, and objectives of, the study clearly described?

Yes

Are sufficient details of the methods provided to allow replication by others?

Yes

Are the datasets clearly presented in a useable and accessible format?

Yes

Reviewer Expertise:

Clinical obstetrics

I confirm that I have read this submission and believe that I have an appropriate level of expertise to confirm that it is of an acceptable scientific standard.
